# Safety-Specific Leadership, Goal Orientation, and Near-Miss Recognition: The Cross-Level Moderating Effects of Safety Climate

**DOI:** 10.3389/fpsyg.2019.01136

**Published:** 2019-05-22

**Authors:** Hongxu Lu, Ting Wu, Yan Shao, Yanbin Liu, Xiaoxuan Wang

**Affiliations:** ^1^School of Business, Ningbo Institute of Technology, Zhejiang University, Ningbo, China; ^2^School of Business, and Research Base of Philosophy and Social Science in Hangzhou—Center for Research of CSR and Sustainable Development, Zhejiang University City College, Hangzhou, China; ^3^Faculty of Economics and Business, University of Groningen, Groningen, Netherlands; ^4^School of Management, Zhejiang University, Hangzhou, China

**Keywords:** safety-specific transformational leadership, safety-specific active transactional leadership, near-miss recognition, learning goal orientation, performance goal orientation, safety climate

## Abstract

Near-miss recognition is an increasingly important area of research in safety management. Drawing on the self-determination theory, we ask whether and how safety-specific transformational leadership and safety-specific active transactional leadership promote near-miss recognition. We also explore the boundary condition by focusing on the moderating role of safety climate. We analyzed time-lagged data from 370 participants, and found that safety-specific transformational leadership enhances employees’ near-miss recognition (by enhancing their learning goal orientation), and that safety-specific active transactional leadership also positively influences employees’ near-miss recognition (by stimulating their performance goal orientation). In addition, we show that safety climate strengthens the relationship between safety-specific transactional leadership and employees’ performance goal orientation, but does not affect the relationship between safety-specific transformational leadership and employees’ learning goal orientation. We discuss the implications and limitations of the research.

## Introduction

Near misses—events in which hazardous conditions could produce a negative outcome but do not (Dillon et al., 2014a)—occur frequently in the workplace (Phimister et al., 2003). As recognizing near misses helps organizations avoid future hazards and improve safety (Kalnins et al., 2006; Kim and Miner, 2007; Soyer and Hogarth, 2015), enhancing near-miss recognition is an increasingly important task in safety management (Baron and Hershey, 1988; Phimister et al., 2003; Soyer and Hogarth, 2015; Dillon et al., 2016). At a Chinese stainless-steel company in 2014, for instance, the failure to recognize a potential workplace hazard led to an explosion, resulting in 146 deaths and 114 injuries. To find ways of promoting near-miss recognition, researchers have begun to explore its antecedents and related contextual factors (Dillon et al., 2013, 2014a; Madsen et al., 2016).

Despite this progress, however, we believe that research on near-miss recognition can be improved in several ways. First, although prior research has confirmed the critical influence of leadership on safety performance (Barling et al., 2002; Kelloway et al., 2006; Inness et al., 2010), it remains unclear whether and how leadership affects near-miss recognition. Second, while much previous research has focused on risk perception as the mechanism explaining the effects of antecedents of near-miss recognition (Madsen et al., 2016), few studies have investigated these effects from the perspective of employees’ motivation. Third, studies have indicated that the relationship between leadership and safety performance is complex and inconsistent (Mullen and Kelloway, 2009; Clarke, 2013; Kark et al., 2015). Further research is needed to explore the factors affecting the relationship between leadership and near-miss recognition. Therefore, our research explores how and when two key safety leadership styles (safety-specific transformational leadership and safety-specific active transactional leadership) influence near-miss recognition.

**FIGURE 1:**
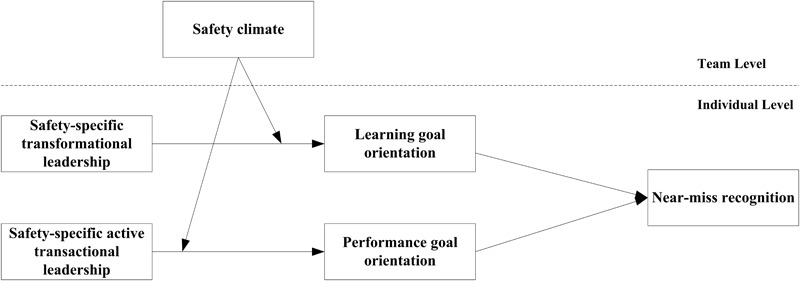
The research model.

Drawing on the self-determination theory (SDT) (Deci and Ryan, 1985; Gagné and Deci, 2005; Deci et al., 2017), we propose that by communicating a positive and value-based vision of the organization to employees, safety-specific transformational leaders enhance near-miss recognition by evoking employees’ learning goal orientation. This orientation reflects the intrinsic motivation to control situations by increasing one’s competence and mastering something new (e.g., Dweck, 1986). We hypothesize that through contingent rewards and management by exception, safety-specific active transactional leaders motivate employees to continuously pay attention to potential safety hazards by fostering their performance goal orientation. This orientation reflects the extrinsic motivation to seek positive feedback or avoid negative evaluations of their competence (e.g., Mehmood et al., 2016). However, the influence of safety-specific leadership on employees’ motivation and safety performance varies with context (Ehrhart and Klein, 2001; Boerner and Freiherr von Streit, 2005). Leaders’ safety-specific behaviors guide employees to pursue safety-related goals, but employees’ safety performance is also influenced by group members’ behaviors and expectations (e.g., Jiang et al., 2010). We propose that the group-level safety climate—i.e., group members’ shared perception of the extent to which safety is valued, supported, and expected within their group (Zohar, 2000; Neal and Griffin, 2006)—moderates the influence of safety-specific leadership styles on employees’ goal orientations. Specifically, a high-safety climate conveys the message that group members value and appreciate safety behaviors, strengthening employees’ motivation to accept and internalize the safety goals advanced by the leader. In contrast, when the safety climate is low, employees may be extremely uncertain about whether the safety behavior advocated by the leader is appreciated and valued by other group members. This weakens the motivational effects of safety-specific leadership. Figure 1 presents our research model.

Our study makes several contributions. First, we respond to the call for leadership studies on near-miss recognition (Dillon et al., 2016) by empirically examining the effects of safety-specific leadership on near-miss recognition. Second, we introduce employees’ motivation as a new theoretical perspective, showing how two important types of safety-specific leadership influence near-miss recognition via learning goal orientation and performance goal orientation. Third, the study provides practical guidance for safety practitioners on enhancing employees’ near-miss recognition by clarifying the particular safety climate under which safety-specific transformational leaders and active transactional leaders can most effectively influence employees’ goal orientation.

## Conceptual Background and Hypotheses

According to SDT, an individual’s performance is affected by intrinsic and extrinsic motivations, which derive from different psychological needs. Specifically, intrinsic motivations drive individuals to engage in activities that satisfy their need for autonomy, while extrinsic motivations inspire individuals to engage in activities that meet their need for competence. In other words, individuals tend to participate in activities and pursue goals that promise to satisfy their key needs. Empirically, previous research has indicated that individuals driven by intrinsic motivations tend to engage in learning activities for personal growth (Ames, 1992), while individuals driven by extrinsic motivations do so to obtain rewards or avoid punishment (Deci and Ryan, 1985). Many studies have supported this view (Wolters et al., 1996; Rawsthorne and Elliot, 1999; Ryan and Deci, 2000; Gagné and Deci, 2005).

### Safety-Specific Transformational Leadership, Learning Goal Orientation, and Near-Miss Recognition

According to SDT, leadership, as an environmental factor, influences employees’ safety performance through their motivations. Safety-specific transformational leadership is a leadership style that delivers a shared vision of safety to employees and encourages them to exercise their energy, skills, and self-efficacy to realize this vision. In practice, a leader with a safety-specific transformational leadership style enhances employees’ safety performance, such as safety participation and compliance with safety regulations, through idealized influence, inspirational motivation, and intellectual stimulation (Barling et al., 2002; Inness et al., 2010). Employees under high level safety-specific transformational leadership, put more energy into safety management and detecting potential safety hazards. Therefore, we propose the following hypothesis:

**Hypothesis 1a:** Safety-specific transformational leadership has a positive effect on employee’s near-miss recognition.

Individuals with a learning goal orientation are likely to adopt in-depth learning strategies (Elliot and McGregor, 2001) and diversified solutions (Bereby-Meyer and Kaplan, 2005) to satisfy their need to understand and master the surrounding environment. They view deficiencies in current safety management as opportunities to improve their ability to master situations in the workplace. Therefore, we speculate that employees with high level of learning goal orientation tend to recognize more potential safety hazards in safety management, enhancing their near-miss recognition.

Safety-specific transformational leadership can affect employees’ safety performance by stimulating their motivation. Research has shown that intrinsic motivations mediate the influence of transformational leadership not only on sports performance (Charbonneau et al., 2001), but also on employees’ creativity (Shin and Zhou, 2003). Some studies on safety management have found that safety-specific transformational leadership is positively associated with employees’ safety compliance via employees’ intrinsic motivations (Conchie and Donald, 2009). Conchie (2013) also found that intrinsic motivations mediate the relationship between safety-specific transformational leadership and employees’ safety citizenship behavior (i.e., voice behavior).

Combining all of the above evidence, and in light of SDT, we argue that safety-specific transformational leadership triggers employees’ intrinsic motivations through idealized influence, inspirational motivation, and intellectual stimulation (Button et al., 1996; Payne et al., 2007). This in turn fosters a learning goal orientation and thus enhances near-miss recognition. Therefore, we propose the following hypothesis:

**Hypothesis 1b:** Employees’ learning goal orientation has a positive effect on their near-miss recognition.
**Hypothesis 1c:** Employees’ learning goal orientation mediates the relationship between safety-specific transformational leadership and near-miss recognition.

### Safety-Specific Active Transactional Leadership, Performance Goal Orientation, and Near-Miss Recognition

Unlike safety-specific transformational leadership, safety-specific active transactional leadership improves employees’ safety performance by clearly conveying contingent incentives and penalties and providing active supervision. Previous research has indicated that safety-specific active transactional leadership can motivate subordinates to obey safety regulations and seek to avoid mistakes and punishment (Wallace and Chen, 2006; Wallace et al., 2008; Kark et al., 2015). When motivated by a leader who provides clear incentives and penalties, employees pay more attention to possible rewards and losses, and thus remain highly sensitive to near misses in safety management. Therefore, we propose the following hypothesis:

**Hypothesis 2a:** Safety-specific transactional leadership is positively associated with employees’ near-miss recognition.

Individuals with a high level of performance goal orientation exhibit more external task motivation and tend to seek more positive and less negative feedback (Wolters et al., 1996; Brett and VandeWalle, 1999; VandeWalle et al., 2001). Individuals with this orientation seek to improve their safety performance to compensate for deficiencies in their work-related ability (Elliot and Church, 1997) and avoid negative feedback (Cron et al., 2005). Therefore, individuals with high level of performance goal orientation are more sensitive to potential safety hazards and thus better able to recognize near misses in safety management. Safety-specific active transactional leadership, an important environmental factor, can affect employees’ safety performance by stimulating their extrinsic motivations. Previous research has shown that employees’ extrinsic motivations mediate the relationship between safety-specific active transactional leadership and employees’ performance (Islam et al., 2012). Studies of safety management have indicated that safety-specific active transactional leadership improves employees’ safety compliance by motivating them to avoid mistakes in safety operations (Kark et al., 2015). Researchers have also found that employees’ extrinsic motivations mediate the relationship between transactional leadership and safety compliance (Pilbeam et al., 2016). Therefore, employees under high level of safety-specific active transactional leadership are better able to recognize near misses because they usually consider near misses as a source of punishment and make more effort to prevent accidents. We thus propose the following hypothesis:

**Hypothesis 2b:** Employees’ performance goal orientation is positively associated with their near-miss recognition.
**Hypothesis 2c:** Employees’ performance goal orientation mediates the relationship between safety-specific active transactional leadership and employees’ near-miss recognition.

### The Moderating Effects of Safety Climate

Drawing on SDT, we anticipate that the effects of safety-specific leadership on employees’ goal orientation depend on employees’ perception of the extent to which safety is valued in their group. This shared perception is also known as the safety climate (Zohar, 2000). Research has suggested that the safety climate varies dramatically across workgroups (e.g., Hofmann and Stetzer, 1996, 1998; Zohar, 2000; Hofmann et al., 2003), and that the strength of the safety climate affects how individuals react to leaders’ behaviors. For example, Hofmann et al. (2003) found that high-quality leader-member exchanges lead to stronger safety citizenship role definitions in a higher-safety climate. Kapp (2012) also suggested that supervisors’ contingent reward leadership practices are more closely related to employee safety compliance or participation when the safety climate is stronger.

When a workgroup has a strong safety climate, members of the group support, value, and appreciate safety practices, such as consistently improving organizational reliability, promoting new security systems, and identifying safety hazards. Therefore, a strong safety climate supports the efforts of transformational leaders, who focus on improving safety performance, and thus enhances their motivational influence. Such a climate also supports the efforts made by active transactional leaders, who focus on minimizing safety hazards in the workplace by adhering to safety regulations, recognizing near misses, and preventing accidents. In contrast, when the safety climate is weak, employees may be extremely uncertain as to whether safety behavior and practices are appreciated and valued by other group members. In such situations, the motivational effects of safety-specific leadership on employees’ goal orientation tend to be weaker. Therefore, we offer the following hypotheses:

**Hypothesis 3a:** The relationship between safety-specific transformational leadership and an individual’s learning goal orientation is moderated by the safety climate, such that the relationship is stronger at high levels of safety climate.
**Hypothesis 3b:** The relationship between safety-specific transactional leadership and an individual’s performance goal orientation is moderated by the safety climate, such that the relationship is stronger at high levels of safety climate.

## Materials and Methods

### Participants and Procedure

We collected data from a large construction company that undertakes various large-scale real estate projects in China and adopted a two-phase time lagged research design. At Time 1, the participating employees reported their perceptions of safety-specific leadership and goal orientation, along with information on their age, gender, educational level, and tenure. One month later, at Time 2, they rated their perceptions of safety climate and near-miss recognition.

We also randomly selected 450 workers from 90 teams on three construction sites. With the assistance of project managers, we asked these workers to fill in questionnaires in the meeting rooms of their respective construction sites during non-working hours. By the end of the survey period, we had received 431 questionnaires at Time 1 (response rate 96%) and 370 questionnaires at Time 2 (response rate 86%). The final matched sample comprised 370 workers (79 teams). The majority of the construction workers were men (88.6%), with a mean age of 38 and a mean tenure of 10.8 years. The participants responded to most of the measures (two types of safety-specific leadership, goal orientation and near-miss recognition) using 5-point Likert scales (1 = “strongly disagree” to 5 = “strongly agree”). We measured perceptions of safety climate on a 7-point Likert-type scale (1 = “totally disagree” to 7 = “totally agree”).

### Measures

We administered all of the questionnaires in Chinese. We translated and back-translated the measures originally developed in English to ensure their validity (Brislin, 1980). The original measurement of near-miss recognition was based on aviation scenarios (Dillon et al., 2016). To tailor it to construction site situations and thus ensure its validity, we took the following steps. First, we created a team consisting of two construction workers with more than 10 years of work experience, two doctoral students with English translation expertise, and two doctoral students in the field of safety management research. Second, this team designed and selected a near-miss scenario on construction sites and developed a questionnaire on near-miss recognition based on previous research (Dillon et al., 2016). Third, we distributed copies of the questionnaire to five experienced construction workers for validation. Based on their feedback, we improved and finalized the scenario and items measuring near-miss recognition.

#### Safety-Specific Leadership

To measure safety-specific leadership, we adopted Conchie (2013) eight-item safety-specific transformational leadership scale and Zohar (2002) six-item safety-specific transactional leadership scale. Sample items for safety-specific transformational leadership include the following: “Our supervisor encourages me to express my ideas and opinions about safety at work” (A = 0.89). The scale for safety-specific active transactional leadership had two dimensions: contingent reward and management by exception (A = 0.91).

#### Goal Orientation

We measured goal orientation using the 16-item scale developed by Button et al. (1996). Eight of the 16 items measured learning goal orientation, and the other eight measured performance goal orientation. For learning goal orientation, a sample item is as follows: “I try hard to improve on my past performance in safety management” (A = 0.90). A sample item for performance goal orientation is as follows: “The things I enjoy the most are the things I do the best in safety management” (A = 0.89).

#### Near-Miss Recognition

Based on Dillon et al. (2016), we developed the following near-miss scenario in the construction context.

*Operating procedures prohibit employees on a construction site from placing nails in their mouths while working, as swallowing nails leads to safety incidents. Due to time limitations and negligence, Mr. Wang placed nails in his mouth during an operation and fell over due to a slippery scaffold. Fortunately, however, the nails in his mouth also fell out and caused no harm*.

After reading the materials, the workers answered a questionnaire evaluating Mr. Wang’s competence, intelligence, and decision-making ability (A = 0.83), and subsequently indicated whether he should be fired (reverse coded). The ICC (1) for near-miss recognition was 0.42, indicating that the sample data we collected in this research were suitable for cross-level analysis.

#### Safety Climate

To measure safety climate, we used Neal and Griffin (2006) three-item scale. Sample items include the following: “Management places a strong emphasis on workplace health and safety” and “Safety is given a high priority by management” (A = 0.72). A series of indicators demonstrated that the safety climate perceived by individual workers could be aggregated to the team level [ICC (1) = 0.25, ICC (2) = 0.59, and median rwg = 0.88].

#### Control Variables

Following previous research (Barling et al., 2002; Inness et al., 2010; Kark et al., 2015), we controlled for demographic variables such as age, gender, education, and tenure. However, the control variables did not change the relationships between the variables of interest and the dependent variables. Thus, for brevity, we report the findings of the analyses without these control variables.

## Results

### Descriptive Statistics

The descriptive statistics and correlations of the variables in our study are presented in Table 1.

**Table 1:** Means, standard deviations, and correlations in individual level.

Variables	*M*	*SD*	1	2	3	4	5	6	7	8	9	10
1. Age	38.00	8.48										
2. Gender	0.11	0.32	0.05									
3. Education	2.25	0.80	-0.42**	0.01								
4. Tenure	10.80	6.14	0.57**	0.09	-0.31**							
5. SSTFL	3.80	0.82	0.08	0.02	-0.13*	0.07	(0.89)					
6. SSATSL	3.70	0.94	-0.03	0.06	0.07	-0.05	0.04	(0.91)				
7. Learning goal orientation	3.87	0.73	0.19**	-0.01*	-0.06	0.11*	0.25**	-0.02	(0.90)			
8. Performance goal orientation	3.86	0.76	0.01	0.12*	-0.04	0.01	-0.00	0.24**	0.04	(0.89)		
9. Near-miss recognition	3.63	0.75	-0.03	0.05	-0.09	-0.05	0.12*	0.14**	0.17**	0.23**	(0.83)	
10. Perception of safety climate	5.56	0.89	0.04	0.01	-0.23**	0.04	0.17**	14^∗∗^	-0.01	0.24**	0.12*	(0.72)

*N = 370. ^∗^*p* < 0.05, ^∗∗^*p* < 0.01. SSTFL, safety-specific transformational leadership; SSATSL, safety-specific active transactional leaders.*

### The Main Effects of Safety-Specific Leadership and Mediating Effects of Goal Orientation

Using Mplus 7.0 (Muthén and Muthén, 2012), we found that both safety-specific transformational leadership (β = 0.11, *p* < 0.05, see Table 2) and safety-specific active transactional leadership were positively related to employees’ near-miss recognition (β = 0.11, *p* < 0.01, Table 2). Thus we found support for Hypotheses 1a and 2a. Further, both learning goal orientation (β = 0.17, *p* < 0.01, Table 2), and performance goal orientation were positively related to near-miss recognition (β = 0.23, *p* < 0.01, Table 2), supporting Hypotheses 1b and 2b.

**Table 2:** The mediation effects of learning goal orientation and performance goal orientation.

Variables	Learning goal orientation	Performance goal orientation	Near-miss recognition
SSTFL	0.22^∗∗^ (0.05)		0.11^∗^ (0.05)
SSATSL		19^∗∗^ (0.04)	0.11^∗∗^ (0.04)
Learning goal orientation			0.17^∗∗^ (0.05)
Performance goal orientation			0.23^∗∗^ (0.05)

	**Mediation effect**	**95% CI of indirect effect, 20,000 bootstrap sampling ^c^**	

SSTFL^a^→LGO→Near-miss recognition	0.04^∗∗^ (0.01)	CI = [0.013, 0.068]	
SSATSL^b^→PGO→Near-miss recognition	0.04^∗∗^ (0.01)	CI = [0.020, 0.074]	

*N = 370, 79 groups. ^∗^*p* < 0.05, ^∗∗^*p* < 0.01. ^*a*^Safety-specific transformational leadership. ^*b*^Safety-specific active transactional leadership. ^*c*^Confidence intervals were calculated using the Monte Carlo method.*

After controlling for safety-specific transformational leadership, we found that as predicted, learning goal orientation was positively related to near-miss recognition (β = 0.04, *p* < 0.01, Table 2). A Monte Carlo based simulation (20,000 repetitions; Selig and Preacher, 2008) revealed that safety-specific transformational leadership enhanced near-miss recognition by stimulating employees’ learning goal orientation (β = 0.04, *p* < 0.01, CI [0.013, 0.068], Table 2), and that safety-specific active transactional leadership positively influenced near-miss recognition by stimulating performance goal orientation (β = 0.04, *p* < 0.01, CI [0.020, 0.074], Table 2). All confidence intervals excluded zero. Thus we found support for Hypotheses 1c and 2c.

### The Cross-Level Moderating Effects of Safety Climate

As shown in Table 3, the influence of employees’ perception of safety-specific transformational leadership on their learning goal orientation (γ = -0.025, n.s.) did not differ significantly between a high-safety climate (+1 SD, above the mean value for safety climate, γ = 0.10, n.s.) and a low-safety climate (-1 SD, below the mean value for safety climate, γ = 0.12, n.s.). In contrast, as hypothesized, safety-specific active transactional leadership interacted with safety climate to predict performance goal orientation (γ = 0.26, 95% CI [0.006, 0.522], Table 3). Simple slope analysis showed that the positive relationship between safety-specific active transactional leadership and performance goal orientation was stronger in a relatively high-safety climate (γ = 0.18, 95% CI [-0.078, 0.438]) than in a relatively low-safety climate (γ = -0.08, 95% CI [-0.216, 0.048], Table 3). These results support Hypothesis 3b but not Hypothesis 3a.

**Table 3:** The cross-level moderation effects of safety climate.

Outcome	Moderator: Safety climate	Effect (*P*_M1X1-W_)	Cross-level moderation effect	95% CI of cross-level moderation effect
Learning goal orientation	Low (-1 SD)	-0.019 (0.11)	0.122 (0.10)	[-0.081, 0.324]
	High (+1 SD)		0.097 (0.11)	[-0.120, 0.315]
	Diff		-0.025 (0.15)	[-0.309, 0.260]
	
	**Moderator: Safety climate**	**Effect (*P*_M2X2-W_)**	**Cross-level moderation effect**	**95% CI of cross-level moderation effect**

Performance goal orientation	Low (-1 SD)	0.203^∗^ (0.10)	-0.084 (0.07)	[-0.216, 0.048]
	High (+1 SD)		0.180 (0.13)	[-0.078, 0.438]
	Diff		0.264^∗^ (0.13)	[0.006, 0.522]

*N = 370, 79 groups. ^∗^*p* < 0.05, ^∗∗^*p* < 0.01. *P*_*M1X1-W*_ refers to the path from the effect of the relationship between safety-specific transformational leadership and learning goal orientation on safety climate; *P*_*M2X2-W*_ refers to the path from the effect of the relationship between safety-specific active transactional leadership and performance goal orientation on safety climate. Diff refers to the cross-level mediation effect difference between safety climate with high level and low level. CI, confidence interval.*

**FIGURE 2:**
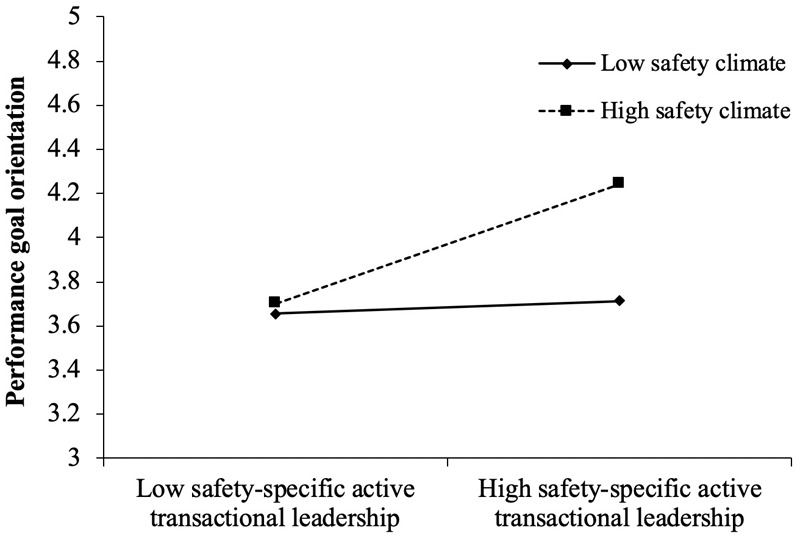
Interactive effects of the safety climate and safety-specific transactional leadership on performance goal orientation.

Using hierarchical linear modeling (Raudenbush and Bryk, 2002; Probst, 2015), we plotted the effect of safety-specific transactional leadership on performance goal orientation in a high- versus a low-safety climate. The plot (see Figure 2) indicated that safety-specific active transactive leadership had a stronger effect on performance goal orientation in a high-safety climate than a low-safety climate.

## Discussion

In this study, we explored how and when safety-specific leadership (i.e., safety-specific transformational leadership and active transactional leadership) enhances employees’ near-miss recognition. The findings suggest that (i) safety-specific transformational leadership enhances employees’ near-miss recognition by stimulating their learning goal orientation, and (ii) safety-specific active transactional leadership enhances employees’ near-miss recognition by promoting their performance goal orientation. Our findings also indicate that safety climate can enhance the positive effect of safety-specific active transactional leadership on performance goal orientation.

However, the results do not support our hypothesis that safety climate moderates the effect of safety-specific transformational leadership on learning goal orientation. This may be due to the scale we used to measure employees’ perceptions of safety climate. All of the items on the safety climate scale, such as “management places a strong emphasis on workplace health and safety,” “safety is given a high priority by management,” and “management considers safety to be important,” stressed the avoidance of safety hazards rather than the improvement of workplace safety. Therefore, we assumed that employees focus on preventing accidents rather than improving the reliability of the organization by mastering new skills related to safety. As a result, we did not observe the expected moderating effect.

### Theoretical and Practical Implications

The findings of this study have several theoretical implications. First, although a growing body of research has explored the antecedents of near-miss recognition (Phimister et al., 2003; Dillon et al., 2013, 2014b, 2016; Soyer and Hogarth, 2015), little attention has been paid to the influence of leadership on near-miss recognition. Extending previous research on the effects of safety-specific transformational leadership and active transactional leadership on employees’ safety performance (e.g., accident reduction and safety compliance), our study for the first time investigated the relationship between safety-specific leadership and employees’ near-miss recognition.

Second, the findings further our understanding of the mechanism by which safety-specific leadership influences employees’ near-miss recognition. Although researchers have studied the relationship between leadership and safety performance from the perspectives of safety climate (Barling et al., 2002; Zohar, 2002; Kelloway et al., 2006), self-regulatory focus (Kark et al., 2015), leader-member exchange (Michael et al., 2006), and trust in leaders (Conchie and Donald, 2009), few have adopted a motivational perspective. Drawing on SDT, we linked employees’ learning and performance goal orientations with different motivations and showed how these orientations mediated the relationship between two safety-specific leadership styles and near-miss recognition. Our study thus provides a new explanation of the relationship between safety-specific leadership and safety performance.

Third, our study highlights the effect of safety climate on the relationship between safety-specific leadership and employees’ goal orientations. Consistent with previous research showing that safety climate can enhance the positive effects and reduce the negative effects of leadership on employees’ safety performance (Zohar, 2002), our findings suggest that a strong safety climate can enhance the effect of safety-specific active transactional leadership on performance goal orientation. They also provide insights into the contingent nature of the relationship between safety-specific active transactional leadership and safety performance.

Additionally, our findings have some practical implications for safety management. First, both safety-specific leadership styles can enhance employees’ near-miss recognition, although the decision on which to adopt depends on the specific workplace conditions. Previous research has indicated that safety-specific transformational leadership and active transactional leadership influence safety performance through different mechanisms (Clarke, 2013). Consistent with this research, our findings suggest that the two types of safety-specific leadership influence employees’ near-miss recognition by prompting different goal orientations. Second, our findings indicate that a positive safety climate can enhance employees’ performance goal orientation but not their learning goal orientation. Thus, for management practitioners, fostering a strong safety climate may enhance the positive influence on safety-specific transactional leadership on employees’ performance goal orientation, but will not affect the link between safety-specific transformational leadership and learning goal orientation.

### Limitations and Future Research

This study has several limitations. First, we only used employees’ self-reported data, which may be subject to common method bias, resulting in false or obscure relationships between variables (Furnham, 1986; Richman et al., 1999). Therefore, future research should collect data from multiple sources by asking supervisors to report on their safety-specific leadership styles or using second-hand data to measure near-miss recognition (Probst, 2015).

Second, because we conducted the study in the construction industry, the average education level in our sample was relatively low, which limits the generalizability of our results. For example, the complicated technology used in nuclear power plants generally requires employees to have a high level of education, so safety climate may have a different moderating effect in the nuclear power industry than in the construction industry. Therefore, we encourage researchers to explore the influence of safety climate on the relationship between safety-specific leadership and goal orientation in a wider range of safety scenarios. In addition, the proportion of women in the construction industry is quite small, which also weakens the generalizability of our results. More research is needed to explore the moderating effect of safety climate on the relationship between safety-specific leadership and goal orientation in safety scenarios with a relatively balanced gender ratio (e.g., in hospitals).

Third, the scale we used to measure safety climate differed from that developed by Zohar (1980). This may explain the insignificant cross-level moderation effect of safety climate on the path from safety-specific transformational leadership to learning goal orientation. Therefore, future research could use the safety climate scale developed by Zohar (1980) for safety management to explore the effect of safety-specific leadership on employees’ goal orientations. The influence of safety-specific leadership on near-miss recognition may also be mediated by other factors (e.g., self-regulatory focus). In this study, goal orientation was the key exploratory variable. We thus call for more research that explores other potential mediators of the influence of safety-specific leadership on employees’ near-miss recognition.

## Conclusion

This study examined how and when two types of safety-specific leadership affect employees’ near-miss recognition. Our results suggest that safety-specific transformational leadership and active transactional leadership positively influence near-miss recognition by stimulating employees’ learning goal orientation and performance goal orientation, respectively. We also found that safety-specific active transactional leadership had a stronger effect on performance goal-orientation when the safety climate was high. However, the positive impact of safety-specific transformational leadership on learning goal orientation was immune to the influence of safety climate.

## Ethics Statement

This study was carried out in accordance with the recommendations of Academic Morality Guidelines by the Academic Committee of Zhejiang University City College, with written informed consent obtained from all subjects. All subjects provided written informed consent in accordance with the Declaration of Helsinki. The protocol was approved by the Academic Committee of Zhejiang University City College. In addition, this study was approved by the Ethics Committee of Zhejiang University City College, and all authors guarantee that the academic ethics was strictly adhered to during the formation of this article.

## Author Contributions

This research is a result of every author. HL mainly proposed the initial idea and basic model for this research, and wrote the introduction part. TW mainly provided his knowledge to perfect the research model and develop the theatrical hypotheses. YS mainly focused on analyzing the data and improving the quality of English. YL and XW mainly contributed to collect and interpret the data of this study.

## Conflict of Interest Statement

The authors declare that the research was conducted in the absence of any commercial or financial relationships that could be construed as a potential conflict of interest.
